# Molecular Characterisation, Tissue Distribution and Pathology of *Circovirus parrot* and *Gammapolyomavirus avis* in Naturally Coinfected Psittacine Birds in Bangladesh

**DOI:** 10.1155/vmi/6646000

**Published:** 2025-12-19

**Authors:** Jannatul Naima, Partha Samanta, Chandan Nath, Md. Sirazul Islam, Md. Saddam Hossain, Pankaj Chakraborty, Subrata Kumar Shil, Md. Ahaduzzaman

**Affiliations:** ^1^ Department of Medicine & Surgery, Chattogram Veterinary & Animal Sciences University, Chattogram, Bangladesh; ^2^ Melbourne Veterinary School, Faculty of Science, University of Melbourne, Parkville, Victoria, Australia, unimelb.edu.au; ^3^ Department of Anatomy & Histology, Chattogram Veterinary & Animal Sciences University, Chattogram, Bangladesh

**Keywords:** *Circovirus parrot*, *Gammapolyomavirus avis*, pathogenesis, molecular pathology, tissue distribution

## Abstract

**Background and Aims:**

*Circovirus parrot* and *Gammapolyomavirus avis* are two clinically important viruses affecting psittacine birds. Although several studies have investigated their genomic variability, pathogenesis and associated pathological features individually, understanding of the effects and pathogenesis of *Circovirus parrot* and *Gammapolyomavirus avis* coinfection remains limited. This study was designed to investigate the molecular characteristics of *Circovirus parrot* and *Gammapolyomavirus avis*, tissue distribution of the viruses and histopathology during the course of the disease in naturally infected birds.

**Methods:**

A total of 16 Circovirus *parrot* and *Gammapolyomavirus avis* polymerase chain reaction (PCR)–positive budgerigars (*Melopsittacus undulatus*) were euthanised, including six without clinical signs and 10 with clinical signs. Ten types of tissue samples were screened using real‐time PCR to detect viruses. Molecular characterisation of the viruses was determined using partial genome sequencing. Additionally, histopathological examination was performed to investigate cellular changes.

**Results:**

Viral distribution varied significantly between tissues (*p* < 0.001) with proportionately higher detection rates in bone marrow (16/16, 100%), cloacal swab (16/16, 100%), feather (15/16, 93.75%) and oral swab (16/16, 100%) for *Circovirus parrot* and in bone marrow (16/16, 100%) and feather (9/16, 56.25%) for *Gammapolyomavirus avis*, than in other tissue types. No significant variation was observed in detection rate between birds with and without clinical signs for both *Circovirus parrot* (*p* = 0.86) and *Gammapolyomavirus avis* (*p* = 0.55). Cellular necrosis and degenerative changes were observed in different tissues, with or without the presence of viral DNA. Molecular analysis suggests that the *Circovirus parrot* circulating in Bangladesh may represent a unique genotype and shares an ancestral relationship with currently circulating strains, whereas *Gammapolyomavirus avis* appears to be less diverse and shares an ancestral relationship with both local and global isolates.

**Conclusion:**

Findings of this study will be useful in understanding the molecular aspects of pathogenesis and disease epidemiology, thus aiding in the design of effective control measures of these diseases.

## 1. Introduction

*Circovirus parrot* is formerly known as *psittacine beak and feather disease virus* (PBFDV) or BFDV. It is a single‐stranded DNA virus of the family *Circoviridae* [1, 2]. The *Circovirus parrot* genome is around 2000 bp long and encodes two major proteins (replication‐associated protein and capsid protein) [3]. The virus infects many bird species globally but is considered one of the most significant contagious and immunosuppressive diseases of budgerigars (*Melopsittacus undulatus*) [2]. Another important contagious‐disease‐causing virus in psittacine birds is *Gammapolyomavirus avis* or *Budgerigar fledgling disease virus* (BFDV), formerly known as *avian polyomavirus 1* (APV‐1), and it is a double‐stranded DNA virus of the family *Polyomaviridae* [4, 5]. The viral genome of *Gammapolyomavirus avis* is around 5000 bp long and comprises two nonstructural proteins (small t and large T antigens) and four structural viral protein genes (VP1–VP4) [6], often found to cause coinfection with *Circovirus parrot* in several parts of the world [7–10]. The *Gammapolyomavirus avis* is more conserved and has a 10‐fold lower evolutionary rate than the *Circovirus parrot* genome [11]. Both viruses are recognised as emerging and re‐emerging threats and have the potential to endanger the parrot species in several parts of the world, thus escalating biodiversity loss and transboundary disease transmission [12]. It is also reported that some parrot species can live a long time asymptomatically with *Circovirus parrot* with or without *Gammapolyomavirus avis* coinfection but may significantly influence disease progression, viral replication, tissue tropism and immune response, complicating diagnosis and management [13, 14]. The viruses can transmit both vertically and horizontally. There is currently no specific treatment for birds infected with *Circovirus parrot* and *Gammapolyomavirus avis*, and these infected birds can act as a source of infection for neighbouring birds. Illegal bird trafficking, lack of quarantine, monitoring and surveillance are important means of horizontal transmission and rapid dissemination of both viruses in naïve areas [15]. Although *Gammapolyomavirus avis* was first detected in captive psittacine birds in Bangladesh in 2023 [9], the detection of *Circovirus parrot* in captive psittacine and nonpsittacine birds was first reported in Bangladesh in 2022 [16].

The *Circovirus parrot* and *Gammapolyomavirus avis* have a particular affinity for mitotically active cells in the beak, feather follicles and immune cells, characterised by epidermal cell necrosis, hyperplasia and hyperkeratosis [8, 17, 18]. Both viruses cause similar clinical signs, manifested by depression, diarrhoea, abdominal distension, dyspnoea and possibly death during the acute phase of the disease in young birds, and abnormal growth and loss of feathers and beak deformities often occur in the chronic phase of the disease [16, 19, 20]. Generally, the dissemination of the virus in tissues is believed to be slower in adult birds and may remain asymptomatic [16, 21]. The tissue distribution and associated pathology have been reported in several studies using conventional polymerase chain reaction (PCR) targeting *Circovirus parrot* in African grey parrot (*Psittacus erithacus*) [22], blue‐fronted parrots (*Amazona aestiva*) [23], Congo African grey parrots (*Psittacus erithacus*) [19] and lorikeets (*Trichoglossus moluccanus*) [24] and *Gammapolyomavirus avis* in budgerigars [25]. However, it was found that pathogenesis and pathology related to *Circovirus parrot* can vary based on genetic variants and bird species [19]. Moreover, little is known about tissue distribution and pathology in *Circovirus parrot* and *Gammapolyomavirus avis* coinfected birds [8, 10, 26].

The purpose of this study was to characterise *Circovirus parrot* and *Gammapolyomavirus avis* using molecular method, to determine the tissue distribution of both viruses and to investigate the associated histopathological findings caused by coinfection in naturally infected birds.

## 2. Materials and Methods

### 2.1. Ethical Approval

The study protocol was reviewed and approved by the Animal Ethics Committee of Chattogram Veterinary and Animal Sciences University (Approval Number: 435(1)/12), following the guidelines for the use of live birds for research purposes.

### 2.2. Study Design and Sampling

All the budgerigars studied were sourced from veterinary clinics, either culled or donated by owners at various times, providing a comprehensive understanding of the disease outcomes and the objectives of this study. In cases where owners had only a pair of birds, with one showing clinical signs and the other without clinical signs, both birds were collected. The birds underwent initial screening using feather samples to detect the presence of *Circovirus parrot* and/or *Gammapolyomavirus avis* [9]. Subsequently, 16 PCR‐positive birds were selected (10 with clinical signs and 6 without clinical signs) and euthanised. Samples from individual birds, including premortem blood (*n* = 16) from the jugular vein collected by an avian veterinarian and postmortem bone marrow (*n* = 16), cloacal swab (*n* = 16), feather (*n* = 16), kidney (*n* = 16), liver (*n* = 16), lung (*n* = 16), oral swab (*n* = 16), skin (*n* = 16) and spleen (*n* = 16) samples, were collected. Blood and swab samples (oral and cloacal) were stored at −20°C and processed for qPCR (*N* = 160). Tissue samples were stored at −20°C for qPCR and at room temperature for histopathology with 10% buffered formalin.

### 2.3. DNA Extraction and qPCR Detection of *Circovirus parrot* and *Gammapolyomavirus avis*

The sample DNA was extracted from individual tissue samples using the Monarch Genomic Purification Kit according to the manufacturer’s instructions (New England Biolabs Inc., USA). The extracted DNA was eluted in a volume of 100 μL. DNA amplification was conducted using SYBR Green assays for *Circovirus parrot* and *Gammapolyomavirus avis*, with the following primers: *Circovirus parrot* (forward: 5′‐CCG​AGA​AGT​ATT​GCA​GTA​AAG​AGG​G‐3′ and reverse: 5′‐TCT​GGG​AAC​TCT​CGC​GCG​AC‐3′) targeting the replication‐associated protein gene (*rep*) and *Gammapolyomavirus avis* (forward: 5′‐GAT​GTG​CAG​AAA​TAG​TGA​GGC​G‐3′ and reverse: 5′‐AGT​GTC​CCG​AGT​GCC​AGA​AG‐3′) targeting the large T antigenic gene (*T-Ag*), as previously described [27]. The qPCR was prepared in a volume of 25 μL, consisting of 12.5 μL of Luna Universal qPCR Master Mix (New England Biolabs Inc., USA), 0.5 μL of each primer (μM), 2 μL of extracted DNA and 9.5 μL of nuclease‐free water. The qPCR thermocycling conditions included an initial denaturation at 95°C for 10 s (s), followed by 40 cycles of denaturation at 95°C for 5 s and annealing and extension at 69°C (*Circovirus parrot* and *Gammapolyomavirus avis*) for 30 s, with a single fluorescence acquisition step at the end of the extension as previously described [27]. Relative quantification for *Circovirus parrot* and *Gammapolyomavirus avis* was determined by the mean cycle threshold (Ct) value considering the established protocol [27]. The qPCR was performed in the Applied Biosystems 7500 Real‐Time PCR System (Thermo Fisher Scientific, USA).

### 2.4. Genome Sequencing and Phylogenetic Analysis

A total of 13 *Circovirus parrot*–positive PCR products and 2 *Gammapolyomavirus avis*–positive PCR products were sent to a biotechnology company (Macrogen, South Korea) for partial sequencing of the *rep* gene of *Circovirus parrot* and the *T-Ag* gene of *Gammapolyomavirus avis*. A lower number of samples for *Gammapolyomavirus avis* were due to the highly conserved nature of the *Gammapolyomavirus avis* genome, thus providing limited variation in phylogenetic analysis as reported earlier [9]. Due to the comparatively small fragment size of the qPCR product (*Circovirus parrot* 142 bp and *Gammapolyomavirus avis* 107 bp) and the NCBI GenBank nucleotide acceptance threshold benchmark (≥ 200 nucleotides), samples were sequenced using a separate set of *Circovirus parrot* and *Gammapolyomavirus avis* primers targeting the same *rep* genes and *large T-Ag*, respectively. The *Circovirus parrot* primers (forward: 5′‐AAC​CCT​ACA​GAC​GGC​GAG‐3′ and reverse: 5′‐GTC​ACA​GTC​CTC​CTT​GTA​CC‐3′) as described by Ypelaar et al. [28] yielded a product of 717 bp. The *Gammapolyomavirus avis* PCR primers (forward: 5′‐CAA​GCA​TAT​GTC​CCT​TTA​TCC​C‐3′ and reverse: 5′‐CTG​TTT​AAG​GCC​TTC​CAA​GAT​G‐3′) were used as described by Johne and Müller [29], yielding a product of 310 bp. Both the forward and reverse sequences of individual sequences were trimmed based on the DNA chromatogram and then aligned, and a consensus sequence was obtained using BioEdit v7. This consensus sequence was then submitted to NCBI GenBank. Similarities between sequences were identified using the NCBI nucleotide BLAST program, resulting in the retrieval of highly similar sequences: 88 sequences for *Circovirus parrot* and 90 sequences for *Gammapolyomavirus avis*. For pairwise distance analysis, relevant sequences were imported to SDT v1.2 and aligned using ClustalW [30]. For phylogenetic analyses, the sequences were imported to MEGA v12, aligned using ClustalW and trimmed to equal length before phylogenetic analysis. The best phylogenetic model was adopted based on model selection analysis incorporated in MEGA v12. The phylogenetic analysis of *Circovirus parrot* was performed using the maximum‐likelihood statistical method, considering the Kimura 2‐parameter model with a gamma distribution for gaps or missing data treatment. For *Gammapolyomavirus avis*, the analysis was conducted using the maximum‐likelihood statistical method, considering the Kimura 2‐parameter model with uniform rates for gaps or missing data treatment. The selection of the model was based on the best‐matched model in MEGA v12. The test of phylogeny was conducted using the bootstrap method with 1000 replications.

### 2.5. Histopathological Examination

An individual tissue sample was fixed in 10% neutral buffered formalin in a container before sending to the histopathology laboratory. Samples were then fixed in paraffin and subsequently were sectioned, mounted, stained with haematoxylin and eosin and examined by light microscopy [31].

### 2.6. Statistical Analysis

Data were analysed using JMP Pro 13 (SAS Institute, USA). Descriptive statistics were employed to summarise the results and presented as positive percentages (%). Analysis of variance was utilised to summarise the qPCR results (Ct values) by tissue type, presenting them as least square means ± standard error, to identify overall significant differences. Tukey’s HSD post hoc test was conducted to assess the significance of differences between group means, and results are presented with different letters (groups not connected by the same letter denote significant differences). A level of *p* ≤ 0.05 was considered statistically significant.

## 3. Results

### 3.1. Characterisation of *Circovirus parrot* and *Gammapolyomavirus avis* Gene Segments

**Figure 1:**
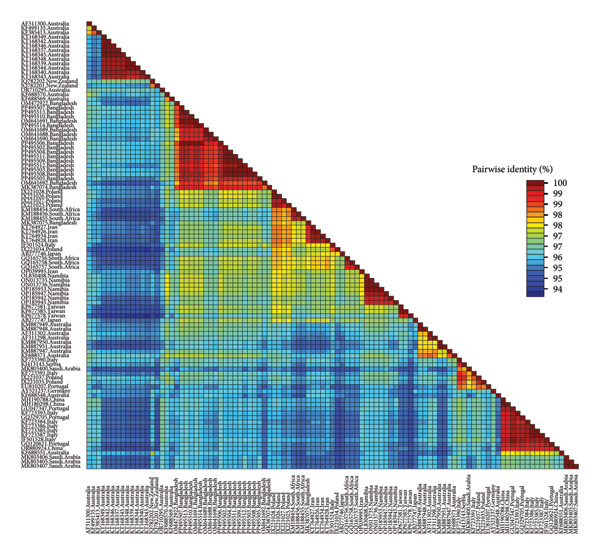
Colour‐coded pairwise identity matrix generated from 13 *Circovirus parrot* of this study (PP495502–PP495514), with other highly similar *Circovirus parrot Rep* gene partial sequences from NCBI GenBank. The matrix was generated using SDT‐1.2.

**Figure 2:**
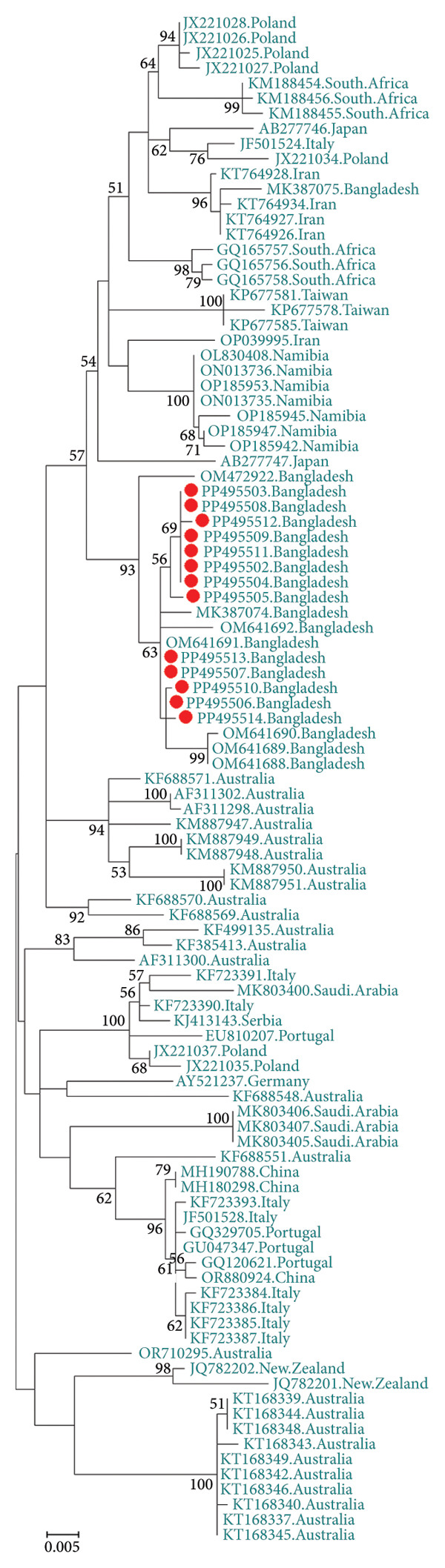
Phylogenetic relationships of the isolated *Circovirus parrot* obtained from naturally infected budgerigars (*Melopsittacus undulatus*) in the Chattogram region of Bangladesh are depicted. Trees are presented with NCBI accession numbers and bootstrap values with 1000 replicates. Sequences from this study are indicated by a red circle.

**Figure 3:**
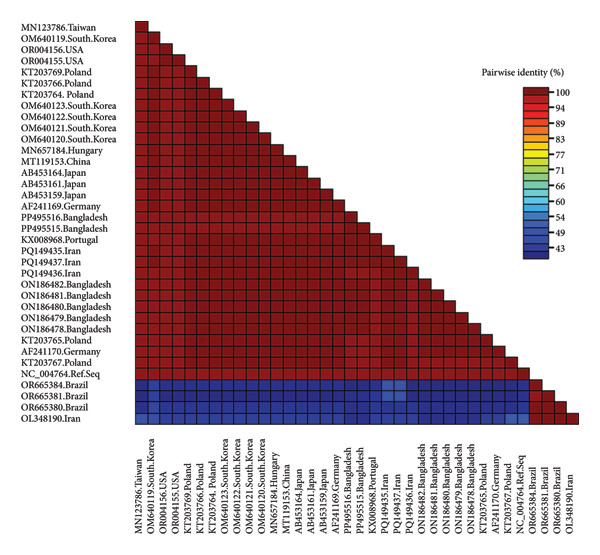
Colour‐coded pairwise identity matrix generated from two *Gammapolyomavirus avis* of this study (PP495515–PP495516), with other highly similar large T antigen gene partial sequences from NCBI GenBank. The matrix was generated using SDT‐1.2.

**Figure 4:**
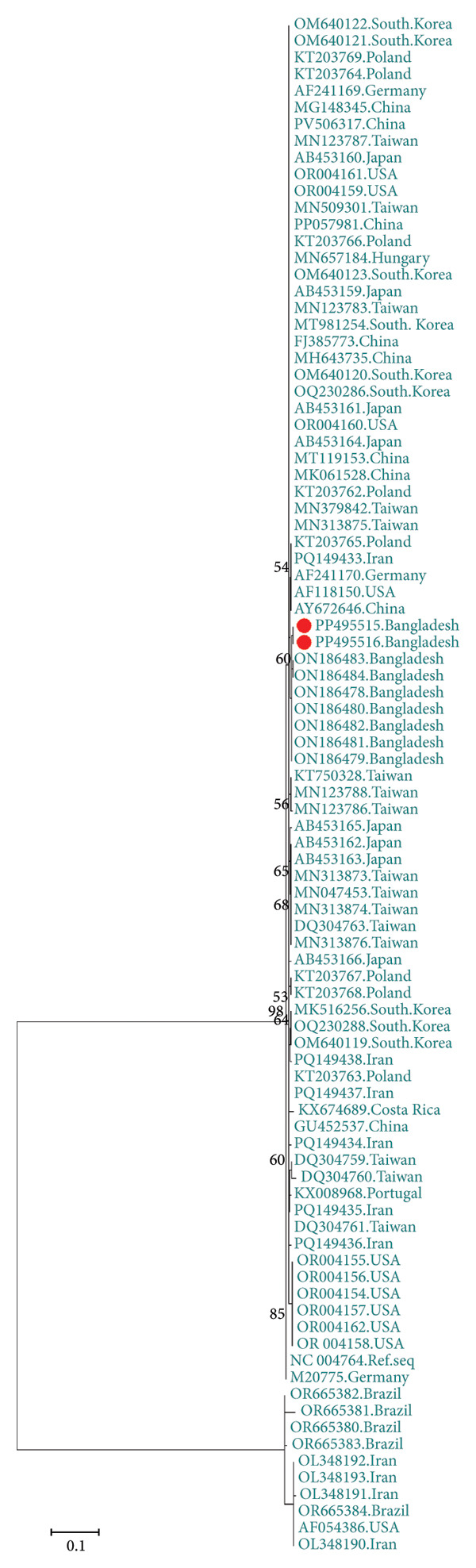
Phylogenetic relationships of isolated *Gammapolyomavirus avis* obtained from naturally infected budgerigars (*Melopsittacus undulatus*) in the Chattogram region of Bangladesh are depicted. Trees are presented with NCBI accession numbers and bootstrap values with 1000 replicates. Sequences from this study are indicated by a red circle.

The GenBank accession numbers of the submitted sequences were PP495502‐PP495514 for *Circovirus parrot* and PP495515‐PP495516 for *Gammapolyomavirus avis.* The *Circovirus parrot* viruses in this study seemed to be a unique genotype and were unrelated to viruses from other geolocations, as depicted based on the nucleotide similarity matrix (Figure 1) and in concordance with phylogenetic analysis (Figure 2). The protein sequence also reflected a similar outcome (Table 1), except for a few sequences from Iran, Poland and Namibia due to silent mutations. The two *Gammapolyomavirus avis* viruses in this study (PP495515–PP495516) showed 99.68% identity using the BLASTN program and showed high nucleotide sequence identity with the selected viruses from other geolocations, including Brazil (except OR665380–OR665384), China, Germany, Hungary, Iran (except OL348190), Japan, Poland, Portugal, South Korea, Taiwan and the United States (Figure 3). Based on protein sequence analysis, the selected *Gammapolyomavirus avis* showed a high level of similarity, except for one sequence from Poland (KT203767) with the point mutation p.D93G and the reference sequence mutations p.S95P and p.E118D (Table 2). Similarly, the phylogenetic tree showed a close ancestral relationship with the previously identified *Gammapolyomavirus avis* from Bangladesh and a distant ancestral relationship with *Gammapolyomavirus avis* from other locations (Figure 4).

**Table 1:** Position of point mutation in the replication‐associated protein (Sites 25–246) of *Circovirus Parrot* in 13 viruses of this study (PP495502–PP495514) and 37 other selected *Circovirus parrot* sequences from NCBI GenBank.

GenBank ID	Geolocation	Amino acid position																						
		25	30	36	47	60	70	72	79	80	82	95	96	97	98	125	133	144	173	198	199	201	207	246
PP495502	Bangladesh	E	L	Y	Q	N	M	P	A	K	S	D	V	I	L	K	D	N	K	D	V	I	G	—
PP495503	Bangladesh	E	L	Y	Q	N	M	P	A	K	S	D	V	I	L	K	D	N	K	D	V	I	G	A
PP495504	Bangladesh	E	L	Y	Q	N	M	P	A	K	S	D	V	I	L	K	D	N	K	D	V	I	G	A
PP495505	Bangladesh	E	L	Y	Q	N	M	P	A	K	S	D	V	I	L	K	D	N	K	D	V	I	G	—
PP495506	Bangladesh	E	L	Y	Q	N	M	P	A	K	S	D	V	I	L	K	D	N	K	D	V	I	G	S
PP495507	Bangladesh	E	L	Y	Q	N	M	P	A	K	S	D	V	I	L	K	D	N	K	D	V	I	G	A
PP495508	Bangladesh	E	L	Y	Q	N	M	P	A	K	S	D	V	I	L	K	D	N	K	D	V	I	G	S
PP495509	Bangladesh	E	L	Y	Q	N	M	P	A	K	S	D	V	I	L	K	D	N	K	D	V	I	G	A
PP495510	Bangladesh	E	L	Y	Q	N	M	P	A	K	S	D	V	I	L	K	D	N	K	D	V	I	G	A
PP495511	Bangladesh	E	L	Y	Q	N	M	P	A	K	S	D	V	I	L	K	D	N	K	D	V	I	G	—
PP495512	Bangladesh	E	L	Y	Q	N	M	P	A	K	S	D	V	I	L	K	D	N	K	D	V	I	G	—
PP495513	Bangladesh	E	L	Y	Q	N	M	P	A	K	S	D	V	I	L	K	D	N	K	D	V	I	G	A
PP495514	Bangladesh	E	L	Y	Q	N	M	P	A	K	S	D	V	I	L	K	D	N	K	D	V	I	G	A
*KT168337*	Australia	E	L	Y	Q	N	M	P	A	K	T	D	**I**	**V**	L	K	D	**S**	K	D	V	I	G	S
*KT168344*	Australia	E	L	Y	Q	N	M	P	A	K	T	D	**I**	**V**	L	K	D	**S**	K	D	V	I	G	S
*KT168346*	Australia	E	L	Y	Q	N	M	P	A	K	T	D	**I**	**V**	L	K	D	**S**	K	D	V	I	G	S
*KT168349*	Australia	E	L	Y	Q	N	M	P	A	K	T	D	**I**	**V**	L	K	D	**S**	K	D	V	I	G	S
*MH190788*	China	E	L	Y	Q	N	**L**	P	A	K	S	D	V	I	L	K	D	N	**R**	**E**	V	**V**	G	P
*MH180298*	China	E	L	Y	Q	N	**L**	P	A	K	S	D	V	I	L	K	D	N	**R**	**E**	V	**V**	G	**P**
KT764926	Iran	E	L	Y	Q	N	M	P	A	K	S	D	V	I	L	K	D	N	K	D	V	I	G	A
KT764927	Iran	E	L	Y	Q	N	M	P	A	K	S	D	V	I	L	K	D	N	K	D	V	I	G	A
KT764934	Iran	E	L	Y	Q	N	M	P	A	K	S	D	V	I	L	K	D	N	K	D	V	I	G	A
*KF723385*	Italy	E	L	Y	Q	N	**F**	P	A	K	S	D	V	I	L	K	D	N	**R**	**E**	V	**V**	G	**P**
*KF723386*	Italy	E	L	Y	Q	N	**F**	P	A	K	S	D	V	I	L	K	D	N	**R**	**E**	V	**V**	G	**P**
*KF723387*	Italy	E	L	Y	Q	N	**F**	P	A	K	S	D	V	I	L	K	D	N	**R**	**E**	V	**V**	G	**P**
*KF723390*	Italy	E	L	Y	Q	N	M	P	A	K	S	D	V	I	L	K	D	N	**R**	**E**	V	**V**	G	**P**
*KF723391*	Italy	E	L	Y	Q	N	M	P	A	K	S	D	V	I	L	K	D	N	**R**	**E**	V	**V**	G	**P**
AB277746	Japan	E	L	Y	Q	N	M	P	A	K	S	D	V	I	L	K	D	N	K	D	V	I	G	A
*AB277747*	Japan	E	L	Y	Q	N	M	P	**S**	**R**	S	D	V	I	L	K	D	N	K	D	V	I	G	A
OL830408	Namibia	E	L	Y	Q	N	M	P	A	K	S	D	V	I	L	K	D	N	K	D	V	I	G	A
ON013735	Namibia	E	L	Y	Q	N	M	P	A	K	S	D	V	I	L	K	D	N	K	D	V	I	G	A
*OP185945*	Namibia	E	L	Y	Q	N	M	P	A	K	S	**E**	V	I	L	K	D	N	K	D	V	I	G	A
ON013736	Namibia	E	L	Y	Q	N	M	P	A	K	S	D	V	I	L	K	D	N	K	D	V	I	G	A
OP185947	Namibia	E	L	Y	Q	N	M	P	A	K	S	D	V	I	L	K	D	N	K	D	V	I	G	A
*JQ782201*	New Zealand	E	L	Y	Q	**S**	M	P	A	K	S	D	V	I	L	K	D	N	**R**	D	V	I	G	**P**
*JQ782202*	New Zealand	E	L	Y	Q	N	M	P	A	K	S	D	V	I	L	K	D	N	**R**	D	V	**V**	G	**P**
JX221025	Poland	E	L	Y	Q	N	M	P	A	K	S	D	V	I	L	K	D	N	K	D	V	I	G	A
JX221026	Poland	E	L	Y	Q	N	M	P	A	K	S	D	V	I	L	K	D	N	K	D	V	I	G	A
*JX221027*	Poland	E	L	Y	Q	N	M	P	A	K	S	D	V	I	P	K	D	N	K	D	V	I	G	A
JX221028	Poland	E	L	Y	Q	N	M	P	A	K	S	D	V	I	L	K	D	N	K	D	V	I	G	A
*GQ329705*	Portugal	E	L	Y	Q	N	**F**	P	A	K	S	D	V	I	L	K	D	N	**R**	**E**	V	**V**	G	**P**
*EU810207*	Portugal	E	L	Y	**R**	N	**N**	P	A	K	S	D	V	I	L	K	D	N	**R**	D	V	**V**	G	**P**
*GU047347*	Portugal	E	L	Y	Q	N	**F**	P	A	K	S	D	V	I	L	K	D	N	**R**	**E**	V	**V**	G	**P**
KM188454	South Africa	E	L	Y	Q	N	M	P	A	K	S	D	V	I	L	K	D	N	K	D	V	I	G	A
*KM188455*	South Africa	E	L	**H**	Q	N	M	P	A	K	S	D	V	I	L	K	D	N	K	D	V	I	G	A
KM188456	South Africa	E	L	Y	Q	N	M	P	A	K	S	D	V	I	L	K	D	N	K	D	V	I	G	A
*GQ165757*	South Africa	E	L	Y	Q	N	M	P	A	K	S	D	V	I	L	K	**H**	N	K	D	V	I	G	S
*KP677578*	Taiwan	E	L	Y	Q	N	M	L	A	K	S	D	V	I	L	**R**	D	N	K	D	**I**	I	**E**	A
*KP677581*	Taiwan	E	L	Y	Q	N	M	P	A	K	S	D	V	I	L	K	D	N	K	D	**I**	I	G	A
*KP677585*	Taiwan	E	L	Y	Q	N	M	P	A	K	S	D	V	I	L	K	D	N	K	D	**I**	I	G	A

*Note:* The sites of mutation in relation to sequences of this study are bolded with italicised GenBank ID. — Missing data.

**Table 2:** Position of point mutation in the T protein (Sites 84–161) of *Gammapolyomavirus avis* in two viruses of this study (PP495515–PP495516), one reference sequence (NC_004764) and 30 other selected *Gammapolyomavirus avis* sequences from NCBI GenBank.

GenBank ID	Geolocation	84	91	93	95	100	110	118	127	137	147	161
PP495515	Bangladesh	G	Q	D	S	Y	D	E	V	I	K	L
PP495516	Bangladesh	G	Q	D	S	Y	D	E	V	I	K	L
ON186478	Bangladesh	G	Q	D	S	Y	D	E	V	I	K	L
ON186479	Bangladesh	G	Q	D	S	Y	D	E	V	I	K	L
ON186480	Bangladesh	G	Q	D	S	Y	D	E	V	I	K	L
ON186481	Bangladesh	G	Q	D	S	Y	D	E	V	I	K	L
ON186482	Bangladesh	G	Q	D	S	Y	D	E	V	I	K	L
OR665380	Brazil	—	—	D	S	Y	D	E	V	I	K	—
OR665381	Brazil	G	Q	D	S	Y	D	E	V	I	K	—
OR665384	Brazil	G	Q	D	S	Y	D	E	V	I	K	—
MT119153	China	G	Q	D	S	Y	D	E	V	I	K	L
AF241169	Germany	G	Q	D	S	Y	D	E	V	I	K	L
AF241170	Germany	G	Q	D	S	Y	D	E	V	I	K	L
MN657184	Hungary	G	Q	D	S	Y	D	E	V	I	K	L
PQ149435	Iran	G	Q	D	S	Y	D	E	V	I	K	—
PQ149436	Iran	G	Q	D	S	Y	D	E	V	I	K	L
PQ149437	Iran	G	Q	D	S	Y	D	E	V	I	K	—
OL348190	Iran	G	Q	D	S	Y	D	E	V	I	K	—
AB453159	Japan	G	Q	D	S	Y	D	E	V	I	K	L
AB453161	Japan	G	Q	D	S	Y	D	E	V	I	K	L
AB453164	Japan	G	Q	D	S	Y	D	E	V	I	K	L
KT203764	Poland	G	Q	D	S	Y	D	E	V	I	K	L
KT203765	Poland	G	Q	D	S	Y	D	E	V	I	K	L
KT203766	Poland	G	Q	D	S	Y	D	E	V	I	K	L
*KT203767*	Poland	G	Q	**G**	S	Y	D	E	V	I	K	L
KT203769	Poland	G	Q	D	S	Y	D	E	V	I	K	L
KX008968	Portugal	G	Q	D	S	Y	D	E	V	I	K	L
OM640122	South Korea	G	Q	D	S	Y	D	E	V	I	K	L
OM640123	South Korea	G	Q	D	S	Y	D	E	V	I	K	L
OM640119	South Korea	G	Q	D	S	Y	D	E	V	I	K	L
OR004155	United States	G	Q	D	S	Y	D	E	V	I	K	L
OR004156	United States	G	Q	D	S	Y	D	E	V	I	K	L
*NC_004764*	Ref. Seq.	G	Q	D	**P**	Y	D	**D**	V	I	K	L

*Note:* The sites of mutation in relation to sequences of this study are bolded with italicised GenBank ID. — Missing data.

### 3.2. Distribution of *Circovirus parrot* and *Gammapolyomavirus avis* in Tissue of Naturally Infected Budgerigars

The *Circovirus parrot* and *Gammapolyomavirus avis* were detected in the bone marrow and spleen of all 16 birds, and blood, liver and skin were negative for both viruses. The cloacal swab was mostly positive for *Circovirus parrot* (15/16, 93.75%) but was negative for *Gammapolyomavirus avis*. Only two kidney samples were positive for *Circovirus parrot*, but none were positive for *Gammapolyomavirus avis* (Table 3). Overall, no significant variation was observed in the detection pattern between birds with and without clinical signs for both *Circovirus parrot* (*p* = 0.86) and *Gammapolyomavirus avis* (*p* = 0.55).

**Table 3:** Tissue distribution pattern of *Circovirus parrot* and *Gammapolyomavirus avis* in 16 naturally infected budgerigars (*Melopsittacus undulatus*), of which 6 birds were without clinical signs and 10 birds were with clinical signs.

Bird ID	Clinical signs	Blood	Bone marrow^$^	Cloacal swab	Feather^$^	Kidney	Liver	Lung^$^	Oral swab	Skin	Spleen^$^
1	FL	−/−	+/+	+/−	+/−	−/−	−/−	+/−	+/−	−/−	+/+
2	NA	−/−	+/+	+/−	+/+	−/−	−/−	+/−	+/−	−/−	+/+
3	NA	−/−	+/+	+/−	+/+	−/−	−/−	+/−	+/−	−/−	+/+
4	NA	−/−	+/+	+/−	+/+	−/−	−/−	+/−	+/−	−/−	+/+
5	NA	−/−	+/+	+/−	+/−	−/−	−/−	+/−	+/−	−/−	+/+
6	A, FL	−/−	+/+	+/−	+/+	−/−	−/−	+/−	+/−	−/−	+/+
7	NA	−/−	+/+	+/−	+/+	−/−	−/−	+/−	+/−	−/−	+/+
8	FL	−/−	+/+	+/−	−/+	−/−	−/−	+/+	+/−	−/−	+/+
9	BD	−/−	+/+	+/−	+/−	−/−	−/−	+/−	+/−	−/−	+/+
10	NA	−/−	+/+	+/−	+/−	−/−	−/−	−/−	+/−	−/−	+/+
11	BD, FL	−/−	+/+	+/−	+/−	−/−	−/−	+/−	+/−	−/−	+/+
12	BD	−/−	+/+	+/−	+/+	+/−	−/−	+/+	+/−	−/−	+/+
13	P	−/−	+/+	+/−	+/−	−/−	−/−	+/+	+/−	−/−	+/+
14	FL	−/−	+/+	+/−	+/−	−/−	−/−	−/+	+/−	−/−	+/+
15	BD	−/−	+/+	+/−	+/+	+/−	−/−	+/+	+/−	−/−	+/+
16	FL	−/−	+/+	+/−	+/+	−/−	−/−	+/+	+/−	−/−	+/+
Total (+)		+0/+0	+16/+16	+16/+0	+15/+9	+2/+0	+0/+0	+14/+6	+16/+0	+0/+0	+16/+16

*Note:* Results are presented as *Circovirus parrot* test results/*Gammapolyomavirus avis* test results. A: ascites; P: paralysis; +: positive; −: negative.

Abbreviations: BD, beak deformity; FL, feather loss; NA, without clinical sign.

^$^coinfected tissue.

The mean *Circovirus parrot* Ct values were higher in lung (20.02 ± 1.28), oral swab (19.99 ± 1.20) and cloacal swab (19.11 ± 1.20), moderate in spleen (18.35 ± 4.97) and bone marrow (17.33 ± 1.20) and lower in feather (13.65 ± 1.24) and kidney (13.90 ± 3.40). Comparing birds with and without clinical signs, a significantly higher *Circovirus parrot* Ct value was detected in lung tissue of birds without clinical signs, whereas a significantly lower Ct value was detected in birds with clinical signs (*p* = 0.025) (Figure 5(a)). Although the Ct values did not vary significantly across tissue types for *Gammapolyomavirus avis* (*p* = 0.21) or with respect to clinical signs (*p* = 0.41) (Figure 5(b)), the mean *Gammapolyomavirus avis* Ct values across different tissues were as follows: bone marrow (29.41 ± 1.76), feather (19.25 ± 3.03), lung (30.11 ± 2.87) and spleen (29.91 ± 4.97). However, a significant variation in Ct values was observed among tissue types for *Circovirus parrot* (*p* = 0.006).

Figure 5The Ct values (LSM ± SEM) of *Circovirus parrot* and *Gammapolyomavirus avis* were measured in 16 naturally infected budgerigars (*Melopsittacus undulatus*), of which six birds were without clinical signs and 10 birds were with clinical signs. The qPCR Ct values were used to express relative Ct values. A higher Ct value indicates a lower copy and vice versa. Levels not connected by the same letter denote a significant difference.

**Figure:**
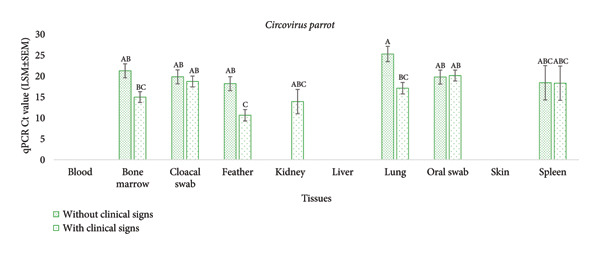
(a)

**Figure:**
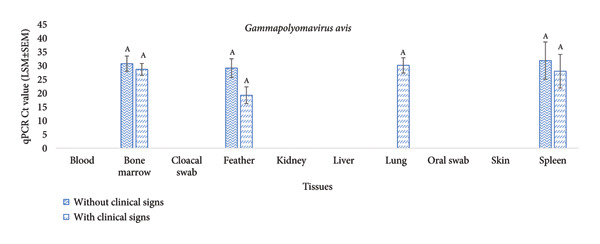
(b)

### 3.3. Histopathological Examination of Tissue Samples From Birds Coinfected With *Circovirus parrot* and *Gammapolyomavirus avis*

**Figure 6:**
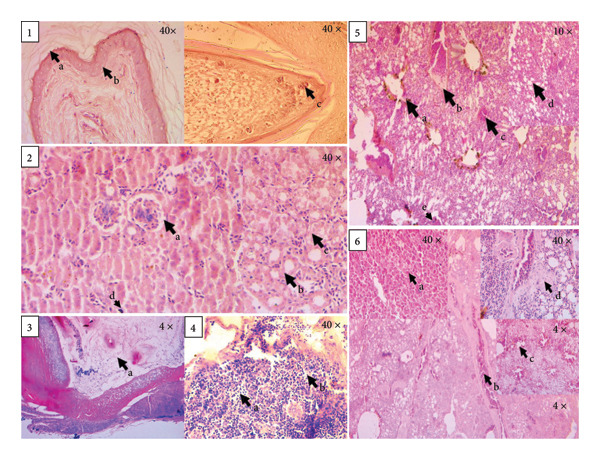
Histopathological changes observed in the skin include hyperkeratosis (1a), ballooning degeneration with a bird’s eye appearance (1b) and haemorrhage and necrosis in feather follicles with epidermal collar necrosis and intracytoplasmic botryoid inclusion body (1c). In the kidney, changes consist of enlarged glomerular lumens (2a), multifocal mononuclear cell infiltration (2b) and interstitial haemorrhage and swelling of tubular epithelium (2c). Bone histopathology reveals abundant infiltration of adipose tissue (3a). Spleen changes include cell necrosis (4a) and disordered spleen forming cavities (4b). Liver alterations show deposition of hemosiderin (5a), oedema and congestion in portal veins (5b), congestion and coagulative necrosis (5c), fatty changes with destruction of hepatic structure (5d) and presence of intracytoplasmic botryoid inclusion body (5e). Lung histopathology presents dense, composed tissue resembling primary atelectasis without alveolar cavities (6a), congested blood vessels (6b), thickened alveolar septa with destruction of alveolar structure and hemosiderin deposition (6c) and fibrosis and round cell infiltration (6d).

The infected birds showed abundant infiltration of adipose tissue in the bone marrow of long bones. The epidermis of the skin exhibited hyperkeratosis, with the presence of a halo zone around the keratinocytes (resembling a bird’s eye appearance), along with pyknotic nuclei and haemorrhage and necrosis in feather follicles, leading to constriction of the feather shaft with the presence of intracytoplasmic botryoid inclusion. In the kidneys, enlarged glomerular lumens were observed, along with swelling of tubular epithelial cells and infiltration of multifocal mononuclear cells and red blood cells with the presence of basophilic globular, botryoid cytoplasmic inclusion bodies. The liver exhibited oedema and congestion in the portal vein, along with hemosiderin deposition, coagulative and punctate necrosis in hepatocytes and infiltration of mononuclear cells and basophilic globular cytoplasmic inclusion bodies. The lungs showed fibrosis and vacuole formation in the alveolar lining, thickening of alveolar septa and hemosiderin deposition, along with congested blood vessels. The spleen exhibited oedematous and necrotic changes, resulting in the formation of cavities (Figure 6).

## 4. Discussion

The study describes the partial genome of *Circovirus parrot* and *Gammapolyomavirus avis* from naturally infected birds, determines viral distribution in different tissues and investigates the associated histopathological changes that occur during infection.

The findings of this study indicate that the *Circovirus parrot* identified here is phylogenetically related to the viruses reported in our previous study [16] but distinct from those detected in other geolocations. Although the evolutionary rate of *Circovirus parrot* is higher than that of *Gammapolyomavirus avis* [11], the newly identified genotype may represent a continuation of the existing Bangladeshi lineage, potentially maintained through the trade of infected birds and subsequent infection by locally circulating strains. Despite recent detections of *Circovirus parrot* in neighbouring countries such as India and Pakistan [2, 5, 32], our findings do not provide evidence of cross‐border transmission, possibly due to the limited sample size. However, *Gammapolyomavirus avis* found here share genetic similarities with viruses from different global regions, suggesting the transboundary transmission of the virus through bird trades. Interestingly, the viruses identified in this study are not closely phylogenetically related to the viruses identified in our previous study in Bangladesh [9], suggesting that divergent genotypes are still emerging in Bangladesh, as in many neighbouring regions [33]. However, complete genome sequencing would be required to strengthen these findings. The lack of virus screening facilities in Bangladesh and quarantine measures raises concerns regarding the possibility of transmission of diverse genotypes of virus to captive populations and native psittacine species. Therefore, more molecular and epidemiological research studies on both *Gammapolyomavirus avis* and *Circovirus parrot* are required to halt their spread and protect the decorative bird breeding sector.

All the birds included in this study were coinfected by *Circovirus parrot* and *Gammapolyomavirus avis*. *Circovirus parrot* was well distributed in almost all tissue types except blood, liver and skin, with a higher viral distribution in feathers, making it an ideal sample for field screening of infection, which is somewhat similar to the findings of a previous study [26]. In contrast, several studies have reported variable patterns of *Circovirus parrot* detection in the blood of different psittacine bird species [34–36]. However, data on budgerigars remain limited, and available studies have mostly yielded negative results [37, 38]. Consequently, it has been suggested that qPCR detection of *Circovirus parrot* in blood samples may not, by itself, be indicative of active infection in psittacine birds unless supported by positive haemagglutination test results [35]. In this study, overall the *Circovirus parrot* relative distribution was comparatively higher (low Ct) than the *Gammapolyomavirus avis* in captive psittacine birds. This could be due to *Gammapolyomavirus avis* infection occurring earlier than *Circovirus parrot* infection, as speculated in the earlier phylogenetic analysis. However, it could also be due to several other reasons such as exposure time, infection dose, virus strains, maternally derived antibodies and viral pathogenesis. A comparatively high viral distribution (low Ct value) was observed in symptomatic birds compared to asymptomatic birds, which is rational, as a higher viral burden is responsible for causing serious damage to body tissues. In this study, we found the tissue distribution of *Gammapolyomavirus avis* in bone marrow, feathers and spleen, with a comparatively higher viral distribution in the feathers using qPCR. Another study reported *Gammapolyomavirus avis* detection from parrots’ livers, but in this study, liver samples tested negative [39]. Similarly, kidney and lung samples were negative in both studies [39]. Another study detected evidence of infection in the kidney and lung of psittacine birds using immunohistochemistry; however, in that study, the majority of birds were *Gammapolyomavirus avis*–negative for these sample types [40]. It has been reported that *Gammapolyomavirus avis* can cause glomerulopathy in nestling nonbudgerigar parrots [41]. The presence of *Gammapolyomavirus avis* in bone marrow and spleen is possibly due to the affinity of virus for lymphoid tissue, as it may cause lymphoid depletion, as observed for goose haemorrhagic polyomavirus, or can cause lymphoma, as observed for canary polyomavirus in a colony of Zebra finches [42]. The pathogenesis of *Gammapolyomavirus avis* and *Circovirus parrot* is not well documented, particularly in coinfected birds, thus warranting further investigation. In this study, the infected birds were not at a stage of viraemia, possibly due to the presence of neutralising antibodies, or the birds were not in the “transit time” stage, or due to the activity of cytokines [43, 44].

Hyperkeratosis and ballooning degeneration of the epidermal layer of the skin, as well as necrosis and congestion in feather follicles, liver, spleen and kidneys, have previously been observed in *Circovirus parrot* and *Gammapolyomavirus avis* infections in cockatiels (*Eclectus roratus*) in Taiwan, which are similar to the findings of this study conducted in budgerigars [45]. Additionally, feather pulp swabs from budgerigars infected with *Circovirus parrot* in Iraq revealed both aseptic and mixed‐cell inflammation, along with the presence of inclusion bodies [22]. The *Gammapolyomavirus avis* is also reported to cause haemorrhage and necrosis in the kidneys and liver at moderate‐to‐low doses of infection and cardiomyopathy at high doses of infection in specific pathogen‐free chicks under challenge infection [18]. The glomerulopathy found in this study is similar to an earlier study [46] and is reported to be due to capillary endothelial damage and immune complex formation [22]. One study reported that *Gammapolyomavirus avis* induces hepatocellular necrosis and intranuclear inclusions are frequently found, especially during the acute stage of infection, and lesions may not be observed microscopically during the chronic stage. In this study, we did not find such inclusion bodies, possibly due to factors such as a lower viral replication rate, immune response, viral latency or persistent low‐level infection, as the birds were chronically infected. Abundant infiltration of adipose tissue in the bone marrow was observed in this study. A previous study reported that excess bone marrow adipose tissue is responsible for causing anaemia and has been reported in a *Gammapolyomavirus avis*–infected fronted parrot (*Amazona aestiva*) [47]. The accumulation of hemosiderin in the liver and lungs could be due to chronic infection, low intestinal pH and supplementation of high‐dietary‐iron‐containing rations under captivity [48, 49]. Fatty changes in the liver are also reported to be associated with chronic infection [50] or the feeding of high‐energy broiler diets to companion birds [51], which could also be reckonable factors in this study.

This study has several limitations. Absolute quantification of *Circovirus parrot* and *Gammapolyomavirus avis* was not possible due to a lack of funding. Additionally, instead of complete genome sequencing, this study presented phylogenetic analysis based on partial genome sequencing due to a shortage of funding.

## 5. Conclusions

All the birds were chronically coinfected with *Circovirus parrot* and *Gammapolyomavirus avis*. The *Circovirus parrot* was genetically diverse and more closely related to previously identified viruses from Bangladesh, whereas the *Gammapolyomavirus avis* from budgerigars showed a phylogenetic relation with viruses from several global regions. Pathological changes were observed in cutaneous and multiple visceral tissues. The highest viral distribution was observed in the feathers of naturally infected birds, hinting at avenues for disease diagnosis and control.

## Ethics Statement

The corresponding author affirms that this manuscript is an honest, accurate and transparent account of the study being reported; that no important aspects of the study have been omitted; and that any discrepancies from the study as planned (and, if relevant, registered) have been explained.

## Disclosure

All authors have read and approved the final version of the manuscript.

## Conflicts of Interest

The authors declare no conflicts of interest.

## Author Contributions

Jannatul Naima: investigation, methodology, data curation, and validation. Partha Samanta: methodology, data curation, and validation. Chandan Nath: conceptualisation, investigation, methodology, validation, and writing–review and editing. Md. Sirazul Islam: investigation and writing–review and editing. Md. Saddam Hossain: conceptualisation and investigation. Pankaj Chakraborty: supervision and writing–review and editing. Subrata Kumar Shil: supervision, methodology, and writing–review and editing. Md. Ahaduzzaman: conceptualisation, investigation, funding acquisition, writing–original draft, and writing–review and editing; visualisation, methodology, formal analysis, software, project administration, supervision, and data curation. All authors had full access to all of the data in this study and take complete responsibility for the integrity of the data and the accuracy of the data analysis.

## Funding

The research was funded by Chattogram Veterinary & Animal Sciences University (CVASU), Bangladesh (Grand Number: 2022‐2023/388/34).

## Data Availability

The *Circovirus parrot* rep gene and *Gammapolyomavirus avis* T antigenic gene partial sequences were deposited in the NCBI GenBank database and were assigned the accession numbers mentioned in the manuscript.
